# Factors affecting women entrepreneurs’ success: a study of small- and medium-sized enterprises in emerging market of Pakistan

**DOI:** 10.1186/s13731-021-00145-9

**Published:** 2021-03-03

**Authors:** Rizwan Ullah Khan, Yashar Salamzadeh, Syed Zulfiqar Ali Shah, Mazhar Hussain

**Affiliations:** 1 Graduate School of Business, Universiti Sains Malaysia, George Town, Malaysia; 2 International Islamic University, Islamabad, Pakistan

**Keywords:** Women, Entrepreneurial success, Risk taking, Need for achievement, Economic factors, Socio-cultural factors, Islamic Republic of Pakistan

## Abstract

In the present era, women are recognized as successful entrepreneurs through their strong desire, qualities, and capabilities for robust economic development. Due to such an important contribution of women in economic development, we propose to investigate the factors which affect women entrepreneur’s success in Pakistan. Data were collected through structured questionnaires from 181 registered SMEs operating in Pakistan. A conceptual model is developed, while SPSS and AMOS software’s are used for analysis. The results indicate that the internal factors including the need for achievements, risk-taking, and self-confidence and external factors including economic factors and socio-cultural factors have a positive and significant influence on the success of women-owned enterprises. This research recommends Small and Medium Enterprises Development Authority (SMEDA), policymakers, and practitioners to encourage women entrepreneurs to run their businesses for the long term by providing a variety of incentives and supports related to those internal and external factors. Numerous studies have been conducted to test the different factors’ effects on women’s entrepreneurial success, but our study investigated some psychological, cultural, and religious factors that are still almost untouched especially in Pakistan. The current study also contributes to the existing literature through empirical shreds of evidence.

## Introduction

Women’s entrepreneurship is a growing global phenomenon, attracting considerable research attention during the last few decades (Henry, Foss, & Ahl, 2016). Not only does it contribute to economies in terms of job creation and economic growth (Kelley, Bosma, & Amoros, 2010), it is also recognized as a source of increasing entrepreneurial diversity in a range of economic contexts (Verheul et al., 2006); as such, it offers a valuable focus for concerted scholarly research. However, despite the significant contribution of women entrepreneurship in Pakistani context, still, it faces numerous barriers and challenges, which can hinder them from entrepreneur’s success (Torres-Ortega, Errico, & Rong, 2015). On the other hand, women entrepreneurs have been ignored to be supported on starting their venture in many emerging economies (Roomi & Parrott, 2008a, 2008b). Unfortunately, less attention has been given to women entrepreneurs in emerging economies despite their sustainable contributions toward GDP (Kelley et al., 2010) and poverty alleviation (Khan, 2014). Due to the complex interaction of socio-cultural factors, religious, and family structures (Roomi, 2013). The role of women in Pakistan’s traditional and masculine society has been the subject of debate. Women face discrimination and gender inequalities owing to gender-biased power relations based on inequality and prejudice (Roomi, Rehman, & Henry, 2018). This research is an attempt to discover factors influencing the performance of women entrepreneurs in this context.

**Fig. 1:**
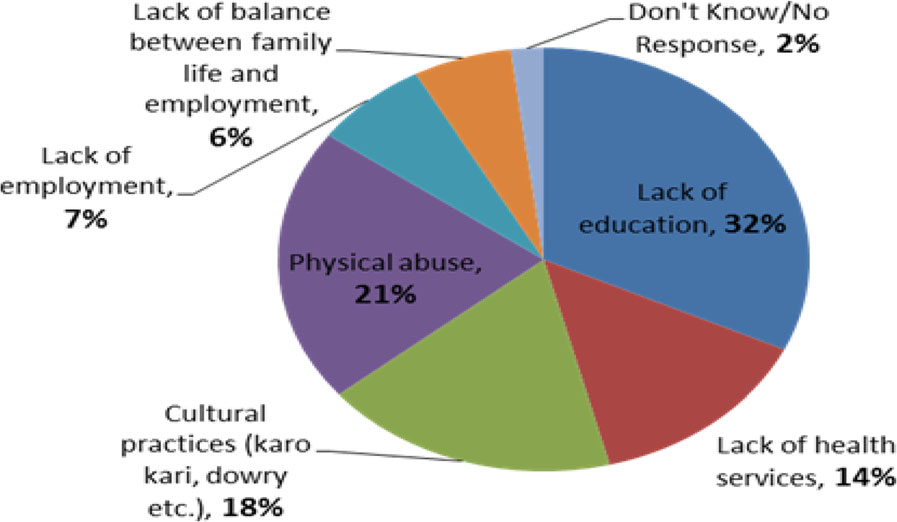
Women entrepreneur’s challenges. Source by Researcher

Therefore, past studies scrutinized that family support, self-confidence and motivation (Azmi, 2017), risk-taking and motivation (Abd Rani & Hashim, 2017), and lack of business skills (Muhammad, McElwee, & Dana, 2017) plays a very important role in women employee performance. While, government policies, access to finance, culture, and regulation (Muhammad et al., 2017) significantly affect women-run enterprises’ success. As (Modarresi, Arasti, Talebi, & Farasatkhah, 2016) finding suggest that employee’s behaviors, culture, economic, and environmental factors significantly affect firm performance. Therefore, the current study examines internal and external factors effect on women firm performance because developing countries have different culture, religious, and cultural activities, which are significantly different from other religions (Khan, 2014); in addition, there is huge uncertainty in economic and government sectors which is a big challenge for women investors during investment decision (Plotnikov, Salamzadeh, Demiryurek, Kawamorita, & Urasova, 2019). Therefore, checking these internal and external factors are very crucial in women’s employee context because sometimes due to government policies or environmental factors, uncertainty can disturb employee behaviors such as motivation, confidence level which negatively affect women’s firm performance. While, definitely several past studies tested these factors in developed countries (Hasan & Almubarak, 2016; Abd Rani & Hashim, 2017) where women entrepreneurs have different supportive rules and regulations for launching their own business and running it. Thereby, Modarresi et al. (2016), demonstrates that both factors can significantly affect women entrepreneurial success in developing economies, underpinning through upper echelons theory (Fig. 1).

In addition, our study suggests several theoretical contributions. In women’s success contexts, many studies have been conducted in few decades but none of them has used upper echelons theory or has not covered all these internal and external factors in emerging economies (Lai, Lin, & Chen, 2017). Upper echelons theory was recognized by Hambrick & Mason, 1984, which demonstrates that the influence of top management behavior personalities affect organizational performance. Here, the current study is conducted on emerging economies while previous studies tested in mixed economies of overall employee performance (Hasan & Almubarak, 2016; Plotnikov et al., 2019; Abd Rani & Hashim, 2017). Nevertheless, there is no single study to check women’s behaviors, attitudes, economic, and environmental factors effect on firm performance in an emerging economy.

### Theoretical background

The main objective of the current study is to determine the effect of the different factors on women’s entrepreneurial success, underpinning through upper echelons theory. It is recognized by Hambrick & Mason, 1984, which explains that the top employee behavior significantly affects organizational performance. In this study, women entrepreneur’s behaviors (self-confidence, risk-taking, and motivation) and external factors significantly affect firm performance. The theory explains that those top managers’ values, behavior, and external factors impact business success. Furthermore, Hambrick (2007) split the upper echelons theory into two parts. The first part explains the external factors (political, environmental, and financial), which influence the entrepreneurial success of the firm.

While the second part explains internal factors such as perceptions and experiences’ impact on top managers of the businesses. Past literature reveals that managers’ traits affect the women-owned business success (Herrmann & Nadkarni, 2014; Heyden, Fourné, Koene, Werkman, & Ansari, 2017). Therefore, we argue that these internal (need for achievement, risk-taking, and self-confidence) and external (economic and socio-cultural) factors are more likely to construct a positive link with business success, according to the upper echelon’s theory.

Several research studies have concluded that managers’ characteristics influence organizations’ strategic behaviors (Colbert et al., 2014; Herrmann & Nadkarni, 2014; Heyden et al., 2017). Thus, it is not surprising that managers with different personalities will prefer different strategic behaviors that will ultimately influence organizational outcomes. There is little doubt that change and innovation in organizations can be brought about by top managers; rather than bottom-line managers (Heyden et al., 2017). Nonetheless, theory development about top management psychological attributes and organization innovation is scarce (Tuncdogan et al., 2017). Hence, research in respect of the upper echelon’s theory is necessary for the current era.

The empirical study focuses on different internal and external factors, which have an impact on women's entrepreneurial success. Hence, define these internal and external factors in their literature as presented in Table 1.

**Table 1:** Main variable’s definitions

S/No	Variable name	Definition	Reference
1	Need for achievement	The concept refers to an individual’s desire for achievement, while it also refers to choose and persist at activities that hold a moderate chance of success or those providing a maximum opportunity of personal achievement satisfaction	Zeffane, 2013; McClelland, 1961
2	Risk-taking	The degree to which managers are willing to make large and risky resource commitments, that is, those which have a reasonable chance of costly failures	Miller & Friesen, 1982
3	Self-confidence	Self-confidence has been defined as that it is the human feeling, which has trust in their qualities, abilities and judgment	Twibell et al., 2008
4	Economic factors	Economic factors refer to the set of basic information related to internal project financing and external market condition that affects the business or an ‘investment’s value	Wube, 2010
5	Socio-cultural factors	These factors include a combination of social and cultural factors that affect women entrepreneurs’ success	Wube, 2010

### Women entrepreneurs in Pakistan

The status of women in Pakistan is not homogenous because of the interconnection of sexual orientation with different types of exclusion in society. Due to cultural norms, religious prescriptions, and practices identified for women, their status and role differ and sometimes is conflicting. These practices enormously restrict the accessibility of opportunities to women all through Pakistan.

Therefore, according to a global entrepreneurship monitor (GEM) (Qureshi, Kratzer & Mian 2011), Pakistan registered in “factors-driven” countries with an early stage total entrepreneurial activity (TEA) rate of 9.07%, at the bottom of the whole group. The early-stage TEA rate for women is 1.73, compared to 15.94 for men. The TEA rate for women is one of the lowest ones among all participating countries in the global entrepreneurship monitor survey (Qureshi, Kratzer & Mian 2011). However, besides all these challenges, the government of Pakistan has started some initiatives for female entrepreneurs and established a women business development center (WBDCs)^1^ in 2012 under the SMEDA, to increase the awareness of women entrepreneurs regarding business start-ups. In addition, the government of Pakistan has started the first women bank Ltd. to help women to get access to loans easier^2^. During 2012, after great women community struggles, they launched Women Business Development Centers (WBDCs) in all big cities of Pakistan^3^.

In this cultural context, women’s participation in business activities creates a big problem with balancing the job and family (Rehman & Azam Roomi, 2012). Moreover, the question becomes more serious when we talk about Pakistan, where women roles, regulations, religious, and culture are different from other nations because Pakistan is a Muslim country, where culture and religion has a vital impact on women profile (Khan, 2014). Hence, the previous studies contribute that women are facing many challenges because of Islamic religious rules and cultural traditions. Similarly, a study conducted in the Iran context, which postulates that women in Islamic developing countries are facing cultural limitations and gender-related inequalities (Modarresi et al., 2016). In addition, Rehman and Azam Roomi (2012) showed that gender bias, lack of time, and family-related issues have a significant impact on women’s business success. Beside all these cultural and religious barriers in Pakistan, there are other problems related to gender as well; for example, women have not full access to the opportunities that men have easy access to (Roomi & Parrott, 2008a, 2008b).

Azam Roomi and Harrison (2010) posit in their literature that;

Women in Islamic countries have barrios to become entrepreneurs. These barriers can be reduced by women having entrepreneurial competencies

Therefore, Modarresi et al. (2016) suggested that women who own businesses are encouraged through intrinsic motivation such as the need for achievement, self-confidence, and socio-cultural activities. While some previous studies also explain that women-owned businesses roles and regulation are significantly different from mixed-gender owned (Rey-Martí, Porcar, & Mas-Tur, 2015). Therefore, female entrepreneurs usually do not invest in a company as much as men, due to less self-confidence level and risk aversion.

## Hypotheses development

### Need for achievement and women entrepreneurs’ success

Achievement is a hidden motivation force developed through the support of human main perception (McClelland, Atkinson, Clark, & Lowell, 1976). It is defined as the desire for success or achievement to excellence (Balogun, Balogun, & Onyencho, 2017) while Jayeoba, Sholesi, and Lawal (2013) defined it as a source of motivation for long-term entrepreneur’s success and an indication for improvement of the desire to get a great achievement in his life or business. Similarly, the willingness to achieve has characteristics such as difficult tasks, responsibility, and focus on success (Rauch & Frese, 2007). McClelland’s motivation theory (McClelland, 1988) suggested that human being has three sorts of achievement motivation needs, need for achievement, need for power, and need for affiliation. However, the need for achievement is essential for top managers to achieve their targets (Dewi, Bundu, & Tahmir, 2016).

Successful entrepreneurs have characteristics such as exploiting opportunities and quick investment decision-making compared to the high market uncertainty (Viinikainen et al., 2017). Nurwahida (2007) claimed that most successful women entrepreneurs have the characteristics of motivation and risk-taking while achievement is also one of the crucial attributes mentioned by Rasheed (2001). Past studies have investigated that those top women entrepreneurs who have high motivation, entrepreneurial intention, and managerial skills can improve their business success easier (Al Mamun & Ekpe, 2016). Moreover, the motivation for achieving objective behavior is supportive for top managers (Rasheed, 2001). Dolan, Peasgood, and White (2008) specified that motivation behavior is not just supporting the managers but at the same time, it plays a vital role as the backbone for achieving their targets.

There is some evidence that women entrepreneurs have a stronger impact on business success, especially in firms that represent relatively fixed personality traits such as motivation (Ehman et al., 2017). While Chuluunbaatar, Ottavia, and Kung (2011) explained that entrepreneurial orientation in the start-up stage affects social capital and personal characteristics, they also have revealed that these own characteristics include motivation and ability of risk-taking. These personal factors also have a positive impact on SMEs (Mahadalle & Kaplan, 2017; Ehman et al., 2017; Chuluunbaatar et al., 2011). Based on the past literature, we propose that women entrepreneurs’ success, who have high motivation and need for achievement, can have a significant and positive impact on their business success.

H1a: The need for achievement has a significant positive effect on women entrepreneurs’ success

### Risk-taking and women entrepreneurs’ success

From an entrepreneur’s success story (Zhang & Cain, 2017), not everyone gets inspired but specifically, those who wish to start their own business, because they want to accept a higher level of risk (Bird, 1988; Chen, Greene, & Crick, 1998). Therefore, previous literature postulated that the risk-taking tendency among entrepreneurs is unfailing (MacCrimmon & Wehrung, 1990). Thereby, the constancy of this idea has confronted from time to time; there are some cross-debates, which believe that entrepreneurs can pose risk-taking capabilities at the same time (Palich & Bagby, 1995). Academic literature postulated that women CEOs in an uncertain situation could take the risk, which significantly affects firm performance and success (Wiklund & Shepherd 2005) because entrepreneurs highly inclined to take risks might receive compensation through higher expected profits (Danso, Adomako, Damoah, & Uddin, 2016).

Women entrepreneurship and risk are two concepts that are viewed as devoted to entrepreneurship literature. For example, women entrepreneurship mostly correlated with risk exposure, separating women entrepreneurship from employees and managers (Begley & Boyd, 1987). For this reason, the way a woman deals with risk is likely to influence the firm’s performance (Pattillo & Söderbom, 2000). Thereby, women entrepreneurs are encouraged to take investment in the turbulent market (Johnell et al., 1995), because female entrepreneurs have the validity to make decisions in the turbulence market (Gedajlovic, Lubatkin, & Schulze, 2004).

Given that, female CEOs are interested in participating in risky activities, while Zalata, Ntim, Aboud, and Gyapong (2019) scrutinized that women are more risk-taker, which significantly impact firm performance and success, especially in emerging economies (Zalata et al., 2019). As such, the level of risk-taking by the women entrepreneur is expected to have a positive impact on performance (Wang & Poutziouris, 2010; Zalata et al., 2019; Zhao, Seibert, & Lumpkin, 2010). Therefore, we postulate from previous literature that women entrepreneurs are more risk-taker oriented during the decision-making process; it, in turn, has some impacts on firm performance and business success.

H1b: Risk-taking has a significant and positive effect on women entrepreneurs’ success.

### Self-confidence and women entrepreneurs’ success

Confidence in entrepreneurship literature is defined as the capability of entrepreneurial perception, which helps entrepreneurs to pursue their target with a strong belief on their way (Twibell et al., 2008). Self-confidence plays a critical role in entrepreneurship literature and it is believed that it helps entrepreneurs in their entrepreneurial activities (Oney & Oksuzoglu-Guven, 2015). While Hassan and Yusof (2015) noted that the self-confidence level of entrepreneurs is their basic thoughts on behalf of their businesses and their interest to face any unexpected failure in the future. Rieger (2012) suggested that entrepreneurs struggle for their objectives with high self-confidence. Abd Rani and Hashim (2017) showed that the women entrepreneurs, who have a high self-confidence level, could quickly gain a competitive advantage in emerging markets while facing different barriers that need to set an objective on or plan a better policy to reach business goals (Moloi & Nkhahle-Rapita, 2014). Similarly, researchers stated that women entrepreneurs, which have a high level of motivation, low anxiety level, and high self-confidence, could better gain a competitive advantage in turbulent markets (Balogun et al., 2017).

On the other hand, entrepreneurial intention is also affected by the self-confidence level and without these factors, it will be impossible to compete in the turbulent markets (Mehtap, Pellegrini, Caputo, & Welsh, 2017). Hence, past literature suggested that women naturally tend to show less intention toward entrepreneurial activities as compared to men, while their decision-making style is also affected by low self-confidence level (Díaz-García & Jiménez-Moreno, 2010). Besides all these issues as mentioned by Al-Dajani and Marlow (2010) due to cultural and religious boundaries in Islamic countries, women mostly get permission from their husband, brother, or father for starting up a new venture; these all can increase or decrease their self-confidence level.

Furthermore, Dabic, Daim, Bayraktaroglu, Novak, and Basic (2012) conducted a study comparing male and female entrepreneurs’ confidence level and found that male entrepreneurs have higher confidence compare to females. Hence, the results from previous literature suggested that women’s entrepreneurial intention and confidence positively influence their business success (Balogun et al., 2017; Mondal, Ghosh, & Das, 2013). Based on the above literature, the below hypothesis is developed:

H1c: Self-confidence has a significant and positive impact on women entrepreneurs’ success*.*

### Economic factors and women entrepreneurs’ success

Economic factors refer to the arrangement of necessary data identified with internal company financing and external financial situation, which influences business success (Wube, 2010). Even though there is an agreement that women can scarcely get credit for their entrepreneurial ventures in many developing countries like Pakistan, the rate of women’s commitment to the economy in the private sector is strikingly contrasted with their male counterparts. In a large number of developing countries, women need to find solutions and gain easy access to finance for their startup businesses. While as mentioned by Afza and Amir Rashid (2009), women entrepreneurs are significantly affected by external factors such as political, financial, and social factors, almost in any sector. Furthermore, Saleem (2017) postulated that women entrepreneurs’ success was significantly affected by external factors such as the environment, government policies, and political issues in emerging economies.

In addition, political, economic, and environmental factors are an external factor and play a pivotal role in firms’ success. In women context, SMEs which have a lack of finance for developmental and other innovative strategies cannot gain and sustain a competitive advantage and manage the political, economic, and social issues themselves (Abdallah & Alnamri, 2015; Radzi, Nor, & Ali, 2017). Lindvert, Patel, and Wincent (2017) found that fluctuation in political and economic systems affects women entrepreneurs’ success. Hence, the external factors (e.g., financial and political factors) significantly increase or decrease business performance and success (Abdallah & Alnamri, 2015; Lindvert et al., 2017; Radzi et al., 2017; Saleem,^ 2017^). Based on the above literature, we propose below hypothesis:

H1d. Economic factors have a significant and positive effect on women entrepreneurs’ success.

### Socio-cultural factors and women entrepreneurs’ success

**Fig. 2:**
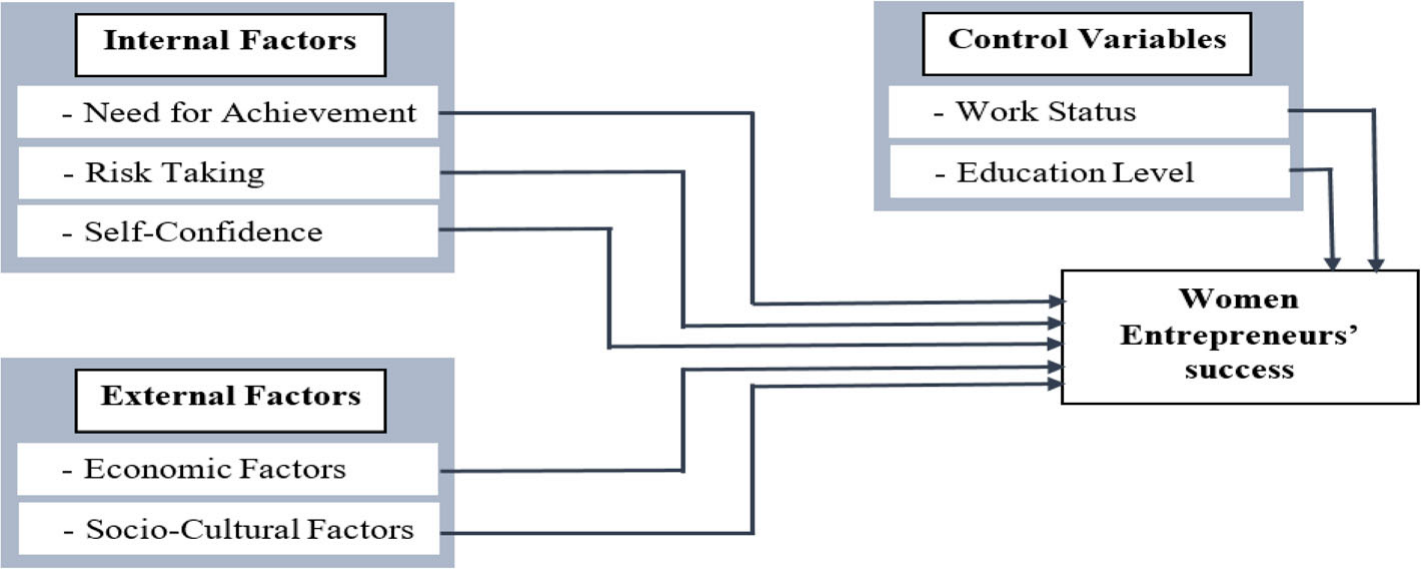
Conceptual framework

Socio-cultural factors include a blend of social and cultural factors that affect women entrepreneurs’ success. In Islamic countries, social and cultural norms on the one hand and family issues, on the other hand, are the most serious issues for women entrepreneurs (Poggesi, Mari, & De Vita,^ 2016^). Hence, Roomi et al. (2018) suggest that women’s entrepreneurial career choices both revolve around and are shaped by a complex interplay of socio-cultural factors. In addition, socio-cultural factors determine the level of entrepreneurial activity in a specific time and place (Veciana, 1999). While Arasti, Zandi, and Talebi (2012) explained that social relationships have a crucial impact on women business’s performance and success. It empowers the business visionary to distinguish opportunities and resources better. Social networks affect entrepreneurs’ business start-up initiatives as it shows them a model for progress and gains support (Mehtap et al., 2017). Even the social bonds and networks with close relatives and life partner is a critical issue for women entrepreneurs’ success (Omwenga, Mukulu, & Kanali, 2013). Balakrishnan and Low (2016) postulated that social-cultural factors (religious, family, etc.) significantly affect women entrepreneurs’ decision-making and success in developing economies. Based on the above-mentioned literature, we propose this hypothesis (Fig. 2):

H1e. Socio-cultural factors has a significant and positive effect on women entrepreneurs’ success.

## Methodology

### Procedure and participants

Data is collected from the SMEs sector operating in the emerging market of Pakistan (Islamabad, Lahore, and Rawalpindi). Only registered SMEs are targeted. The list of registered SMEs is taken from the Islamabad Chamber of Commerce, Lahore chamber of commerce, and Rawalpindi chamber of commerce, verified by Small and Medium Enterprises Development Authority (SMEDA), which is a governmental institute under the Ministry of industries and production, established in October 1998. The target population are women SMEs, definition in Pakistani context, those small enterprises which have more than 10 and less 250 employees SMEDA, 2018; Khan, Salamzadeh, Kawamorita, & Rethi, 2020), managed and controlled by women. We collected data from 3 April 2020 to 15 July 2020 through structured questionnaires from women entrepreneurs. There is a total of 323 women-owned small enterprises registered in these three big cities run and managed by women directors. For selection of sample, we used (Krejcie and Morgan, 1970) sample calculation table because we have total firm detail taken from SMEDA. For choosing the sample, we used simple random sampling technique because sampling random sampling techniques is widely used techniques in small enterprises (Healey, 2014). In random sampling techniques, every sample or enterprise has an equal chance (Kumar, 2008). For selection the total number of sample, Krejcie and Morgan, table, we distributed 323 questionnaires through email and Google doc. Approach to women entrepreneurs and finally, because of Covid-19, we cannot personally visit the enterprises due to governmental restriction of SOPs. Hence, after distribution, we received 197 questionnaires. From this number of questionnaires, 16 questionnaires had some missing values and therefore excluded from our final samples. Hence, a total of 79 responses from Rawalpindi, 54 from Islamabad, and 48 from Lahore are used in this research.

#### Measurement instrument

In the current study, we adapted the scales of main constructs from the previous literature and used confirmatory factor analysis for checking the reliability and validity of the main constructs.

#### Women entrepreneurs’ success

The women entrepreneurs’ success is dependent variable and it is measured through the nine-item questionnaire adapted from previous literature used by Maehr and Sjogren (1971) by crown back alpha more than 0.70.

#### Self-confidence

The self-confidence is an independent main construct that refers to the employee’s motivation and encourage toward the achievement. Self-confidence is measured through a six-item questionnaire adapted from the previous studies developed by Jones, Swain, and Cale (1991) with more than 0.70 crown back alpha.

#### Need for achievement

Next, need for achievement is an independent main construct, which it refers to the employee’s struggling for achieve the main objective. In the current study, need for achievement measured through adapted previous studies using a five-item questionnaire developed by (Zeffane, 2013) having crown back alpha more than 0.70.

#### Risk taking

Furthermore, risk-taking is a third independent main variable; it refers to the employee’s bold decision for gaining the external opportunity. In the current study, risk-taking is measured through 4 items adapted questionnaires from previous studies developed by Steensma and Corley (2001) having more than 0.70 crown back alpha.

#### Economic factors

The fourth one is an economic factor, and in the current model, it plays a role as independent main construct. The economic factors refer to the different economical aspects that effect on the firm and economic stability. It is measured through adapted questionnaires from previous literature using a four-item developed by Wube (2010) having crown back alpha more than 0.70.

#### Socio-cultural factors

The fifth one is a socio-cultural factor, and in the current model, it plays a role as independent main construct. The socio-cultural factor refers to the different external cultural which is related to an employee’s traits effect on firm’s and economics’ instability. It is measured through adapted questionnaires from previous literature using a four-item developed by Wube (2010) having crown back alpha more than 0.70.

### Control variables

For the purpose to reduce spurious insights of this study, we used work status and women education level as control variables to check different factors effect on women’s entrepreneurial success. Because Huarng, Mas-Tur, and Yu’s (2012) finding postulate that women entrepreneurs status plays a significant role in firm performance while on the other side, educated women take good investment decisions that impact firm performance (Robinson, Blockson, & Robinson, 2007). Therefore, we suppose these two variables to control the firm performance.

### Common bias method

Subsequently, collecting data through a single source (questionnaire), the common bias method (CBM) issue might happen (Podsakoff & Organ, 1986). Women cannot easily take decision, so that is why their every strategic decision are full of biases. Therefore, for checking CBM, we applied Harman’s one-factor using factor analysis by extraction method of “principal component analysis” in SPSS. Harman’s one-factor test results show that there are six factors, which have Eigenvalues more than 1, while their first factor is explaining 32.45% of the total variance. It indicates that there is no such a kind of common bias method problem in the data, as the first factor explains a great part of the total variance (Hair, Anderson, Babin, & Black, 2010; Podsakoff & Organ, 1986).

### Measurement model results

The current study uses AMOS for measurement model, validity, reliability, normality, and multi-collinearity for fruitful insights. Table 2 explains that data normality is assessed through the skewness and kurtosis; the results are in the acceptance rage ± 2, which explains that observed variables of this study are within the normal range (George & Mallery 2010; Khan & Ghufran, 2018). In addition, in the current study, we evaluate the Mardia’s coefficient using wed basd software “https://webpower.psychstat.org/models/kurtosis” suggested by Cain, Zhang, and Yuan (2017) because we collected the data from women top managers, so it needs to check the deep normality. Hence, the results show that skewness and kurtosis values are 1369.008 and 3080.546 respectively with *p* value < 0.05, which is larger than Mardia’s coefficient (Mardia, 1970) (see Table 4 for more detail). Hence, on the base of Mardia’s results, we reject the null hypotheses because the data are not normally distributed. While, the current study shows that there is no multicollinearity problem in the model because the latent variables have a variance inflation factor (VIF) less than 3 (Hair et al., 2010) and tolerance value is greater than 0.10. Hence, it indicates that there is no multi-collinearity problem in the model as shown in Table 3 (Hair et al., 2010).

**Table 2:** Demographics Results

Description	Frequency	Cumulative percent
Marital status		
Single	85	46.96
married	96	53.03
Work status		
Full time	137	75.69
Part-time	44	24.30
Education level		
Intermediate and less	28	15.47
Bachelor	63	34.80
Master	49	27.07
MS/MPhil	32	17.67
PhD	9	4.97
Business size		
20–50 employees	67	37.02
51–100 employees	52	28.73
101–150 employees	27	14.91
151–200 employees	23	12.71
201 to 250 employees	12	6.63
Total	181	100

**Table 3:** Mean, S.D, and normality

	WEO	MSC	MNFA	MRT	MEF	MSCF
Mean	3.74	3.72	3.67	3.37	3.77	3.78
Std. Deviation	0.37	0.28	0.40	0.35	0.36	0.31
VIF	–	1.490	1.565	1.340	1.225	1.141
Tolerance	–	0.671	0.281	0.299	0.449	0.467
Skewness	− 0.355	− 0.308	− 0.064	− 0.106	− 0.849	− 0.084
Kurtosis	0.336	0.022	0.235	0.216	1.905	0.102

Women entrepreneurial success, self-confidence, risk taking, need for achievement, economic factors, socio-cultural factors

**Fig. 3:**
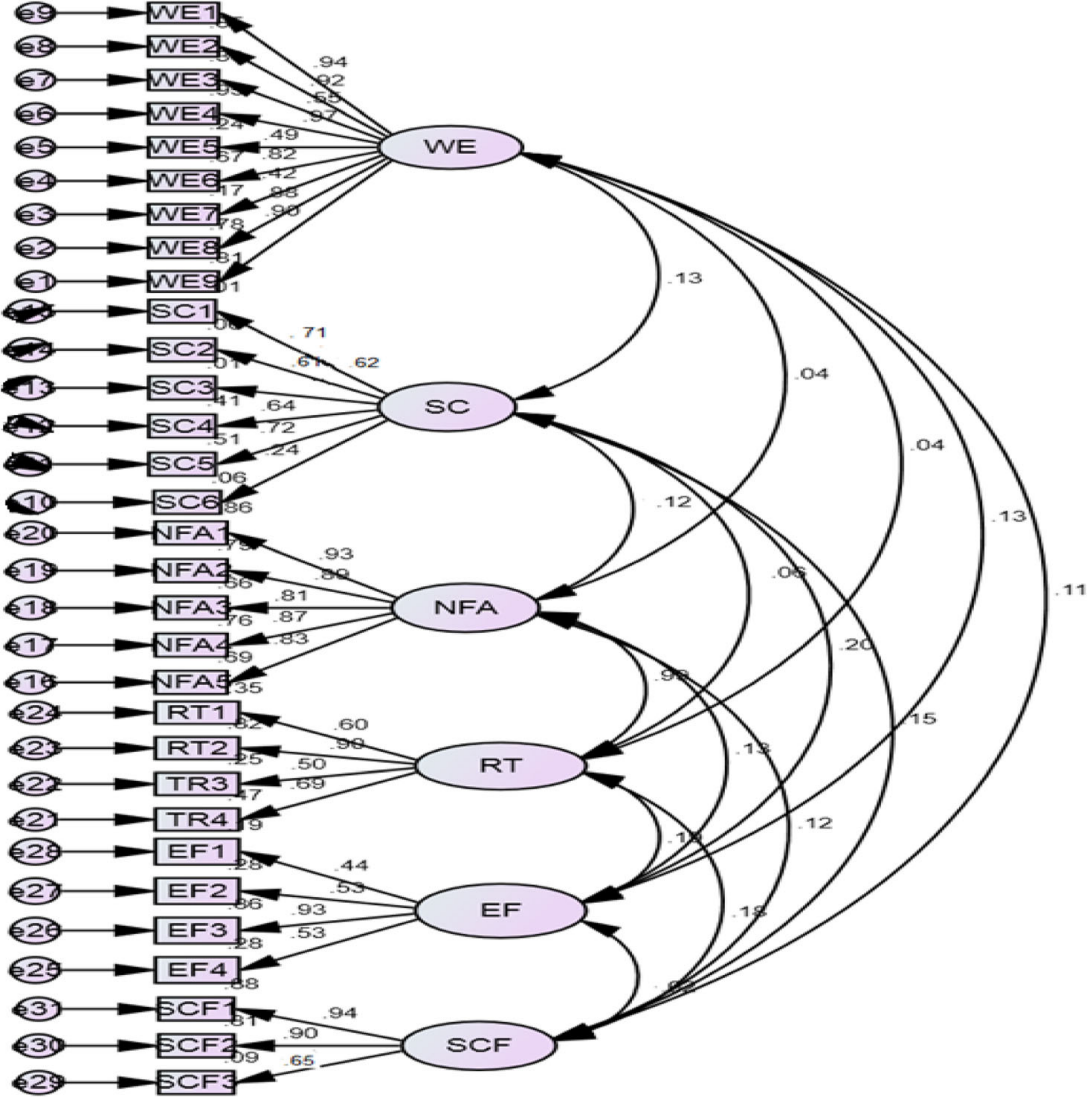
Confirmatory factor analysis

A confirmatory factor analysis contended through AMOS to check the model fitness, reliability, and validity as shown in Fig. 3. All the items factor loading above the threshold level of 0.70 and significantly loaded (*p* < 0.01) of all their respective items.

The convergent validity is identified by taking a square of all items errors in factor loading on a construct and then is divided to the sum of all factor loadings on the average number of items. The results in Table 5 explain that all constructs have convergent validity of more than 0.50 (Hair et al., 2010; Khan, 2019). Discriminant validity is calculated by taking the square root of AVE and results are shown in Table 4. The discriminant validity results interpret that each value of the discriminant validity value is more than 0.70, which shows that all items are unique as required (Hair et al., 2010). In addition, composite reliability indicates the loaded internal stability of each construct. In Table 5, the results are shown and it is clear that all constructs have reliability of more than 0.70, which is the accepted range as recommended by Nunnally and Bernstein (1994).

**Table 4:** Mardia’s multivariate skewness and kurtosis

	*b*	*z*	*p* value
Skewness	1369.008	73,013.76394	0
Kurtosis	3080.546	87.85813	0

**Table 5:** Standardized factor loadings, validity, and reliability

Contracts	Estimate	AVE	C.R
Women entrepreneur success			
There is an increase in sales and profitability during the three last years	0.899**	0.51	0.77
My business does not have the capacity to develop new products and processes	0.885**		
I do not think that my business will survive and continue its activity forever	0.417*		
I am committed with social responsibility, i.e., employing local nationals	0.820**		
My business is offering high-quality products and services	0.485**		
More than 50% of the profit are reinvested in the business	0.974*		
Profits of my enterprise tend to increase	0.546**		
The number of employees in my enterprise started to increase	0.920**		
There is an increase in sales and profitability during the three last years	0.944**		
Self-confidence			
I feel at ease	0.737**	0.50	0.70
I am confident to perform well in his business take care	0.717**		
I am concerned about these competitors in the market	0.743*		
Our team needs to perform well.	0.611**		
Our team is physically and mentally well prepared to complete the opposition in the market	0.623**		
Never my team workers feel nervous during hard working	0.714**		
Need for achievement			
I always do my best whether I am alone or with someone	0.832**	0.67	0.87
I always try hard to improve on my past performance	0.874*		
I enjoy working towards clear, challenging goals	0.815**		
In general, I try to make every minute count	0.891**		
I often put pressure on myself to achieve as much as I can	0.927**		
Risk-Taking			
We seem to adopt a rather conservative view when making major decisions	0.686**	0.51	0.72
We tend to support projects where the expected returns are certain	0.701**		
Operations have generally followed the "tried and true" paths	0.904**		
Our operations can be generally characterized as high risk	0.595**		
Economic factors			
My business is not offering a good product at a competitive price	0.534**	0.50	0.71
I can’t find qualified labor for my business	0.926*		
There is the maintenance of accurate records of sales/expense in my enterprise	0.533**		
I am satisfied with the financial facilities given by banks and other lending institutions	0.840**		
Socio-cultural factors			
I suffer greatly from the gender discrimination caused by the culture and traditions of the society	0.652**	0.65	0.83
The support from strong ties (spouse, parents, friends and relatives) have a positive effect on my business growth	0.899**		
My parents have their own business	0.939**		

Significant *p < .005; **p < 0.001

The first test of model fitness results was not good; therefore, we checked the model modification indices (MI) and the results indicate that there are some redundancy items in our model. Therefore, we removed problematic items and the second round of results shows a good model fit.

Thus, the model fit criteria achieved are shown in Table 6. However, Barclay, Higgins, and Thompson (1995) suggested that we need to ensure the validity and reliability of the constructs before testing hypotheses in structural equation modeling. Table 6 shows a good model fit result according to confirmatory factor analysis (CFA).

**Table 6:** Model fit indexes

Fitness criteria	Measurement model	Acceptable range^a^
CMIN	237.270	–
DF	156	–
CMIN/DF	1.520	1–3
GFI	0.95	> 0.90
AGFI	0.84	> 0.80
CFI	0.97	> 0.95
TLI	0.91	> 0.90
NFI	0.93	> 0.90
RMR	0.023	< 0.09
RMSEA	0.010	< 0.08
P CLOSE	0.083	> 0.05

^a^No single rule for model fit. This study followed Hair et al. (2010) criteria that mostly cited and recommended in prior studies

### Correlation

In the current study, we used Pearson correlation by SPSS software for evaluating the correlations. The results indicate that there is a significant positive relationship between self-confidence and women entrepreneurs’ success (*r* = 0.39, *p* < 0.05). Furthermore, we found that the need for achievement has a significant positive correlation with women entrepreneurs’ success (*r* = .30, *p* < .001) and similarly risk-taking also has a significant positive correlation with women entrepreneurs’ success (*r* = 0.01, *p* < 0.43). While economic factors have a significant positive correlation with women entrepreneurs’ success (*r* = 0.522, *p* < 0.001) and finally socio-cultural factors have a significant positive correlation with women entrepreneurs’ success (*r* = 0 .41, *p* < 0.05) (Table 7).

**Table 7:** Correlation and reliability

Contracts	Items No.	1	2	3	4	5	6
Women entrepreneur success	9	(**0**.**77**)					
Self-confidence	6	0.39^a^	(**0**.**70**)				
Need for achievement	5	0.29^b^	0.559^b^	(**0**.**87**)			
Risk-taking	4	0.43^a^	0.505^a^	0.831^a^	(**0**.**72**)		
Economic factor	4	0.52^b^	0.180^a^	0.184^a^	0.238^b^	(**0**.**71**)	
Socio-cultural factors	3	0.41^a^	0.078^a^	0.145^b^	0.162^a^	0.726^b^	(**0**.**83**)

^a^ Significant at the 0.05 level. ^b^ Significant at the 0.01 level

Table 8 represents the multiple regression model, as the current model has no depending factors, which affects the model therefore the current relationship just reveals between predictor and response variables. Thereby, our results explain that the need for achievement has a positive and significant impact on the women entrepreneurs’ success (β = 0.25, *p* value > 0.05); it suggests that women managers having a strong motivation and intention to achieve his target in the Pakistani context. Therefore, it explains that women entrepreneurs’ success enhances 25% if there is a 1% increase in women entrepreneurs’ motivation to get their targets.

**Table 8:** Multiple regression

Model	Unstandardized coefficients		Standardized coefficients	*t*	Sig.	*R*^2^	Δ*R*^2^
	B	Std. Error	Beta				
Step 1							
Work status	0.173	0.035	0.134	2.240	0.026		
Education level	0.158	0.158	0.236	4.194	0.000	0.016	0.016
Step 2							
Work status	0.173	0.034	0.134	2.673	0.015		
Education level	0.158	0.037	0.236	4.287	0.000		
Need for achievement	0.254	0.057	0.271	4.509	0.000		
Risk taking	0.288	0.031	0.437	3.313	0.005		
Self-confidence	0.645	0.062	0.482	7.878	0.000		
Economic Factors	0.243	0.069	0.253	3.516	0.002		
Socio-cultural factors	0.340	0.038	0.498	8.928	0.000	0.191	0.175

Dependent variable: women entrepreneurial success

Moreover, our result reveals that risk-taking has a significant and positive impact on women entrepreneurs’ success (β = 0.29, *p* value > 0.00). It shows that if women entrepreneurs can take decisions (for example, financial or investment decisions) wisely having all the barriers in their minds, it can push their business toward success in the long term. Therefore, it means that for each 1% increase in taking a risky decision, a 29% increase in their business success will be expected.

In addition, self-confidence has a positive and significant relationship with women entrepreneurs’ success (β = 0.64, *p* value > 0.05). It shows that the women entrepreneurs, who have a high self-confidence level, take any uncertainty issue very easily without feeling any depression, so it can help during firm performance and success for the long-term. It means that for each 1% increase in self-confidence level, there will be a 64% increase in women entrepreneurs’ success.

In external factors, our results explain that economic factor has also a significant and positive impact on women entrepreneurs’ success (β = 0.24, *p* value > 0.05). It explains that if there is no uncertainty such as political, environmental, or financial in a business environment, then firms can achieve their targets smoothly, so we found that women entrepreneurs are significantly affected by these factors, because each 1% fluctuation in external factors, its effects on women entrepreneurs’ success will be 24%.

Similarly, socio-cultural factors also have a significant impact on women entrepreneurs’ success (β = 0.34, *p* value > 0.05); it postulates that women entrepreneurs have many socio-cultural factors in their business environment; they cannot achieve their targets because these factors significantly contribute on their success. Hence, the underline study explains that each 1% interchange in these factors in their business environment will result in a 34% impact on their success.

As *R*^2^ = 0.191 in our research model, it indicates that 19% of the total variance in women entrepreneurs’ success can be using these internal and external factors such as the need for achievement, self-confidence, risk taking, economic factors, and socio-cultural factors.

## Discussion and conclusion

Results of the current study reveal that internal factors including self-confidence, risk taking, and need for achievement, and external factors including economic and socio-cultural factors have a positive and significant impact on women entrepreneurs’ success in Pakistan. So, our findings support the previous studies results in both developed and developing economies such as Azmi (2017), Abd Rani and Hashim (2017), and Muhammad et al. (2017) suggested that women entrepreneurs internal behavior self-confidence and motivational force enhance the competitive advantage; these capabilities help them to become a successful women entrepreneur. On the other hand, some other researchers (Hasan & Almubarak, 2016; Muhammad et al., 2017) suggested that external factors also influence women entrepreneurs’ success. While, our study is different from developed economies finding because they (Bastian, Sidani, & El Amine, 2018; Laudano, Zollo, Ciappei, & Zampi, 2018) suggested that these factors cannot significantly enhance women entrepreneurs’ success.

The current study concluded that the “need for achievement” factor has a positive and significant impact on women entrepreneurs’ success. So, our findings are consistent with previous studies (Chuluunbaatar et al., 2011; Mahadalle & Kaplan,^ 2017^; Ehman et al., 2017) who suggested that women entrepreneurs who have a high level of motivation can succeed during the business. Hence, our finding demonstrates that those women entrepreneurs, who have a high degree of motivation for working or starting a new venture, can easily success in his job. While Bastian, Sidani, & El Amine (2018), who conducted a study in the Middle East, suggested that motivation for gaining his objective can play a pivotal role in business success. So, on the base of aligning our results with previous studies, our H_1_ is supported.

Next, our results postulate that risk-taking has a positive and significant impact on women entrepreneurs’ success. Therefore, our finding favors past literature (Meroño-Cerdán, López-Nicolás, & Molina-Castillo, 2018) who demonstrated that feeling hesitation during decision-making can affect the firm performance. In addition, Panno, Donati, Milioni, Chiesi, and Primi (2018) suggest that new ventures have many taking risk behaviors as compare to old firms. Therefore, our findings are aligned with previous studies that women entrepreneurs who feel risk-aversion will affect their SME’s performance and success (González, Guzmán, Pombo, & Trujillo, 2013; Panno et al., 2018).

Self-confidence is very essential for top managers during decision-making for the long and short term. Therefore, our findings postulate that self-confidence has a positive and significant influence on women entrepreneurs’ success in emerging economies. Because our finding is familiar with previous studies conducted in developed economies (Balogun et al., 2017; Mondal et al., 2013), which suggest that top management with high confidence can easily compete in the market and get success. While Oney and Oksuzoglu-Guven’s (2015) finding demonstrates that bold manager can easily take investment decision as compared to having low confidence. Therefore, on the basis of these arguments, we suggest that confidant women entrepreneurs can easily get success. However, we differentiate our study, as the context of it is an Islamic economy, where rules and regulations are different for women to achieve success in their business. Therefore, our study suggests that women entrepreneurs who work in an Islamic context and still take self-confidential decisions are having a great impact on their SMEs’ performance and success.

In the underline study, we argue that internal factors have a positive and significant impact on women entrepreneurs’ success, while external factors also have a positive influence on women entrepreneurs’ success. Thereby, our findings are consistent with previous studies, which are conducted in developed economies (Radzi et al., 2017) who suggest that finance plays a vital role in SME’s success. While Lindvert et al. (2017) suggested that political interference is very important for networking with customers and suppliers. Therefore, on the basis of this past literature, we posit that external factors are significantly contributing to women entrepreneur’s success in emerging economies. In addition, our findings suggest that socio-cultural factors have a positive and significant impact on women entrepreneurs’ success. Therefore, our results favor past studies (Arasti et al., 2012; Poggesi et al., 2016) who demonstrate that social and cultural factors have a positive and significant impact on women entrepreneurs’ success. While Roomi et al. (2018) suggest that in an Islamic country, women entrepreneurs face a lot of cultural conflicts related his business because in Islamic cultural, women are following their roles and regulation. Therefore, we posit in the underline study that these factors significantly affect the women entrepreneur’s success.

## Contribution of the study

### Theoretical contribution

The underline study contributes to the existing literature in the field of women entrepreneurial success, internal factors (motivation, risk-taking, and self-confidence), and external factors (economic and socio-cultural). The main objective of the underline study is to examine the internal and external factors, which influence women’s entrepreneurial success. Our study finding acknowledges that several researchers investigate women entrepreneurial performance through different factors, but the current study evaluates the effect of the internal and external factors on women small and medium-sized enterprises in developing economies because more than 70% of SMEs are operationalized in developing economies. Therefore, their culture, religion, and regulation are different from a developed country. Therefore, our current study underpins through the “upper echelons theory” Hambrick & Mason, 1984; it explains that the top manager’s behaviors and external factors impact on business performance. Because in our model, motivation, risk-taking, and confidence are internal entrepreneurial behaviors and economic and social factors are external factors, which enhance the business performance in Pakistani small-medium enterprises.

### Practical implications

The current study not only provides the implications to manager and owners of SMEs but also gives guidelines to policymakers and particularly support the institution such as SMEDA. Our findings demonstrate that women entrepreneurs need motivation and confidence to start their business by arranging the seminar, workshop, women incentives, or women entrepreneurial university. Because our results suggest that if women have motivation and confidence, it can enhance entrepreneurial performance. Therefore, we recommend to government and policymaker to arrange a seminar or women entrepreneurial university, which helps the women entrepreneurs to create courage and start a business. In addition, according to Hussain, Mahmood, and Scott (2019), out of 97 small business in Pakistan, only 5% are women entrepreneurs; so, we suggest to policymakers and government to give the education about the business and incentive to starts his own business because SMEs have 40% contribution in Pakistani GDP.

### Limitation and future direction

There is no study without limitation, and ours has some limitations as well. The main limitation is related to our sampling, which has been done in three large cities, but future researchers can expand it to a national level to reach more comprehensive results with some considerations on geological and cultural differences. So, we recommend for future researchers to relate the current work with different Islamic countries (comparative study). The current finding suggests to the future researcher that uses a mixed-method approach among women entrepreneurs in developed countries. It is also suggested to consider more variables in future researches to get a bigger image of this research idea. Finally, yet importantly, testing any moderator such as literacy or financial literacy, which increases or decreases the relationship between these factors and women entrepreneurs’ success, is also suggested.

## Data Availability

We collected the data from small medium and enterprises of various cities of Pakistan. Please note that we can also avail of the do-files upon request.

## Abbreviations

SMEs: Small- and medium-sized enterprises
SMEDA: Small Medium Enterprises Development Authority
GDP: Gross domestic products
GEM: Global entrepreneurship monitor
WBDC’s: Women Business Development Center

## Footnotes

1 https://www.pakistanpressfoundation.org/women-business-development-centre-inaugurated/

2 http://www.fwbl.com.pk/assets-products/business-loans-for-women/

3 https://smeda.org/index.php?option=com_content&view=article&id=236&catid=8&Itemid=101
